# The Removal of an Incarcerated Kuntscher Nail in a Patient Presenting With Femoral Neck Fracture: A Technical Case Report

**DOI:** 10.7759/cureus.89323

**Published:** 2025-08-04

**Authors:** Dimitrios Kalatzis, Styliani-Despoina Christidi, Konstantinos Kaoullas, Anna Lenti, Androniki Drakou

**Affiliations:** 1 Orthopedics and Traumatology, Laiko General Hospital of Athens, Athens, GRC

**Keywords:** femoral nail extraction, incarcerated femoral nail, incarcerated nail, kuntscher nail, nail removal

## Abstract

The Kuntscher nail, once a pioneering solution for femoral shaft fracture fixation, has largely fallen out of use with the emergence of modern interlocking intramedullary systems. However, retained Kuntscher nails may still be encountered in older patients and can pose significant technical challenges when removal is required, especially in cases of incarceration. Standard closed extraction methods are often ineffective, necessitating alternative surgical strategies. This case report describes a novel and practical technique for the successful extraction of an incarcerated Kuntscher nail in a patient requiring hip arthroplasty following a femoral neck fracture. A 68-year-old male presented with a left femoral neck fracture after a fall. Imaging revealed a retained Kuntscher nail from a femoral shaft fracture treated 32 years earlier. Initial attempts at closed nail extraction through a trochanteric incision using standard tools were unsuccessful due to nail incarceration. A transverse femoral osteotomy was subsequently performed at the level of the nail shaft, and the nail was transected using a high-speed pneumatic drill. The distal nail segment was removed through the osteotomy site, while the proximal segment was extracted via the original incision. The procedure was completed with a hemiarthroplasty and open reduction internal fixation (ORIF) of the osteotomy. The patient had an uneventful postoperative recovery, with satisfactory osteotomy healing and preserved hip function at the one-year follow-up.

Incarcerated Kuntscher nails often resist removal through conventional closed extraction techniques. Surgeons should be prepared to employ alternative strategies when standard methods fail. This case report presents a novel and practical technique for the successful extraction of a retained Kuntscher nail, offering a valuable option in such challenging scenarios.

## Introduction

Interlocking intramedullary nailing remains the current gold standard for the treatment of femoral diaphyseal fractures, offering superior biomechanical stability and earlier mobilization compared to older fixation methods [1]. However, before the widespread adoption of interlocking systems, the Kuntscher nail played a pivotal role in revolutionizing the management of long bone fractures. Introduced by Gerhard Kuntscher during World War II, the cloverleaf-shaped intramedullary nail represented a significant advancement in fracture care, allowing for internal splinting of the femur with minimal surgical exposure [2]. Although initially met with skepticism, the technique gained rapid acceptance, particularly in military settings, due to its success in enabling early mobilization and return to function.

The Kuntscher nail functions as a rigid device placed within the medullary canal, providing axial stability. However, unlike modern interlocking nails, it relies solely on a press-fit mechanism or locking bolts distally, which can lead to incarceration due to bone overgrowth and endosteal callus formation over time. As such, removal of the Kuntscher nail, especially after decades in situ, can pose a substantial technical challenge. In current orthopedic practice, younger surgeons may have limited exposure to this implant, and its removal is rarely required. Nevertheless, retained Kuntscher nails may still be encountered, particularly in older patients presenting with new pathology, such as fractures or joint degeneration, requiring surgical intervention. In such scenarios, nail extraction becomes a prerequisite for definitive management, such as hip arthroplasty.

This case report presents the surgical management of a 68-year-old male with a femoral neck fracture and a retained, incarcerated Kuntscher nail from a previous femoral shaft fixation performed 32 years earlier. We describe a practical and effective technique involving a transverse femoral osteotomy and the use of a high-speed pneumatic drill to facilitate nail removal, followed by hemiarthroplasty and internal fixation. Our report highlights the importance of preoperative planning and intraoperative adaptability in the successful treatment of complex revision cases involving legacy orthopedic implants.

## Case presentation

**Figure 1:**
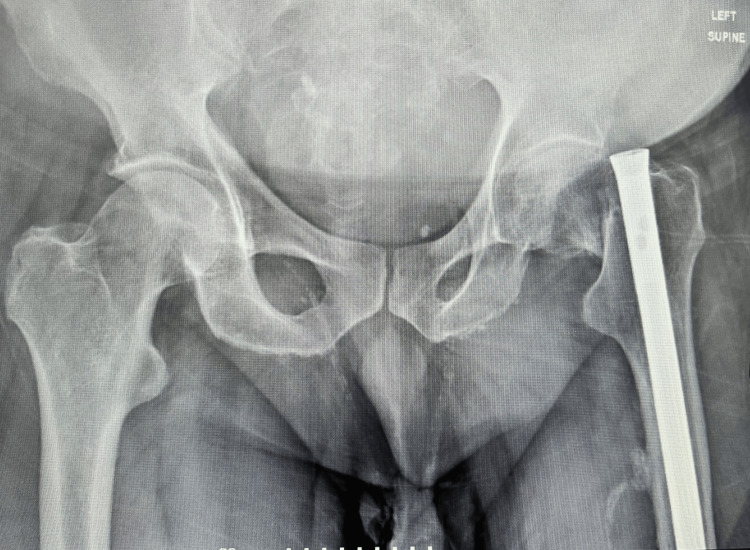
Preoperative radiograph showing a femoral neck fracture of the left hip in a patient with a retained Kuntscher nail from a previous femoral shaft fracture fixation

A 68-year-old male presented to the emergency department after sustaining a fall onto his left hip. Radiographic evaluation confirmed a femoral neck fracture. The patient had a history of a left femoral shaft fracture treated 32 years prior with intramedullary fixation using a Kuntscher nail (Figure 1). According to the patient’s history and radiographic findings, the shaft fracture had healed uneventfully. Plain radiographs revealed three cerclage wires at the previous fracture site and the presence of a Kuntscher nail secured with two distal locking screws. Neurovascular examination of the affected limb was unremarkable.

Given the anticipated difficulty of extracting a Kuntscher nail, thorough preoperative planning was essential. Our primary plan involved attempting nail removal using standard extraction tools, including hooks and a single guide wire with a beveled, angled distal end [3]. If successful, this would be followed by a hemiarthroplasty to address the femoral neck fracture. As an alternative, and a more time-consuming approach, we prepared for a transverse osteotomy at the level of the femoral shaft. This technique involved cutting the nail using a high-speed pneumatic surgical drill, facilitating the removal of the nail in two segments. Following extraction, a hemiarthroplasty would be performed in conjunction with open reduction and internal fixation (ORIF) of the osteotomy site.

In the operating room, the patient was positioned in the lateral decubitus position. The procedure began with the removal of the distal locking screws. The proximal femur and Kuntscher nail were accessed through the previous trochanteric incision. An extraction tool and hook were engaged at the proximal end of the nail; however, despite multiple attempts, the nail remained firmly incarcerated.

**Figure 2:**
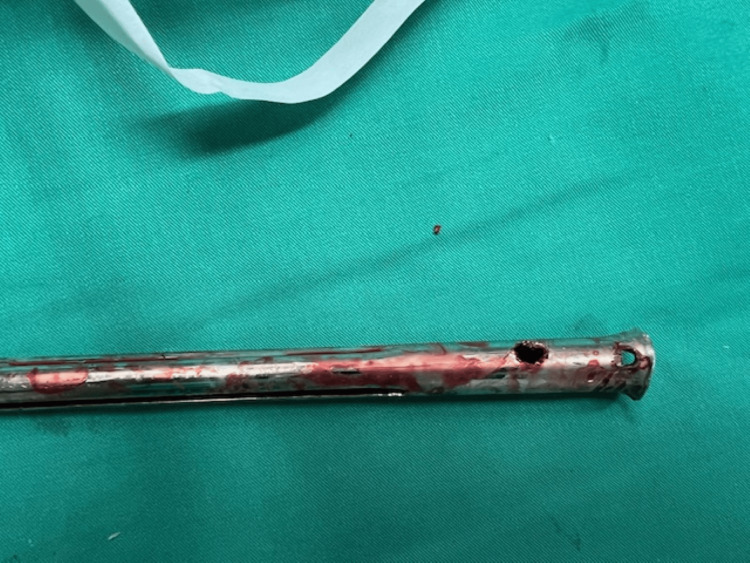
Proximal segment of the Kuntscher nail after the extraction

**Figure 3:**
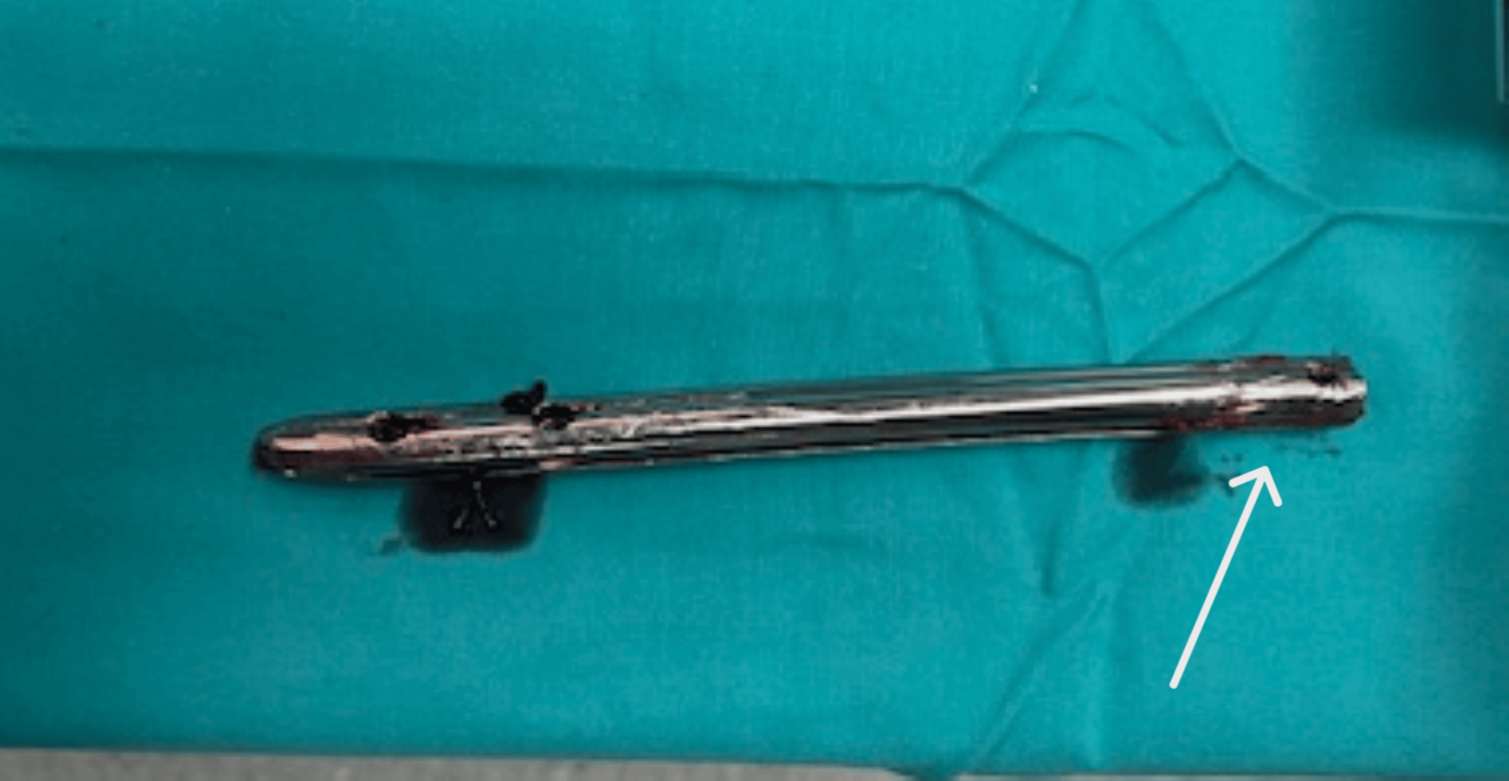
Distal segment of the Kuntscher nail after the extraction Channel created using a pneumatic drill (white arrow)

**Figure 4:**
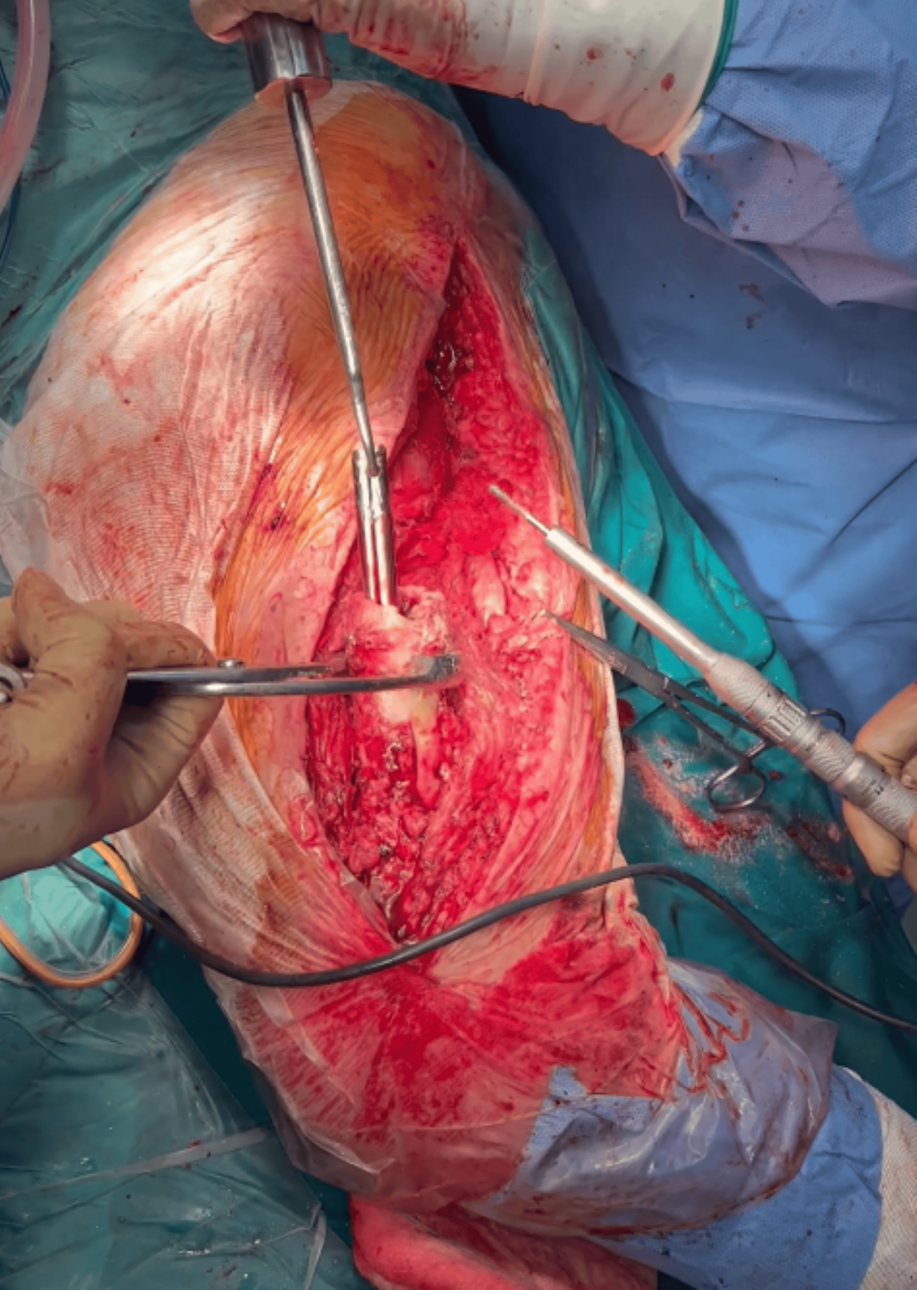
Intraoperative photograph showing the extraction hook engaged in the channel created within the distal segment of the Kuntscher nail to facilitate removal

Given the failure of closed extraction, we proceeded without any delay to the alternative surgical plan. A transverse osteotomy of the femoral shaft was performed using a high-speed pneumatic surgical drill, along with a transverse cut of the nail at the same level. The existing cerclage wires were also removed at this stage. Varus angulation of the osteotomy was applied to facilitate access to the now-separated proximal and distal nail segments. The distal portion of the nail was extracted through the osteotomy site. The proximal segment was pushed proximally through the osteotomy and retrieved via the original trochanteric incision (Figure 2). To aid in the removal of the distal nail fragment, a channel was created using the pneumatic drill (Figure 3), allowing for the secure engagement of a hook used for extraction (Figure 4).

**Figure 5:**
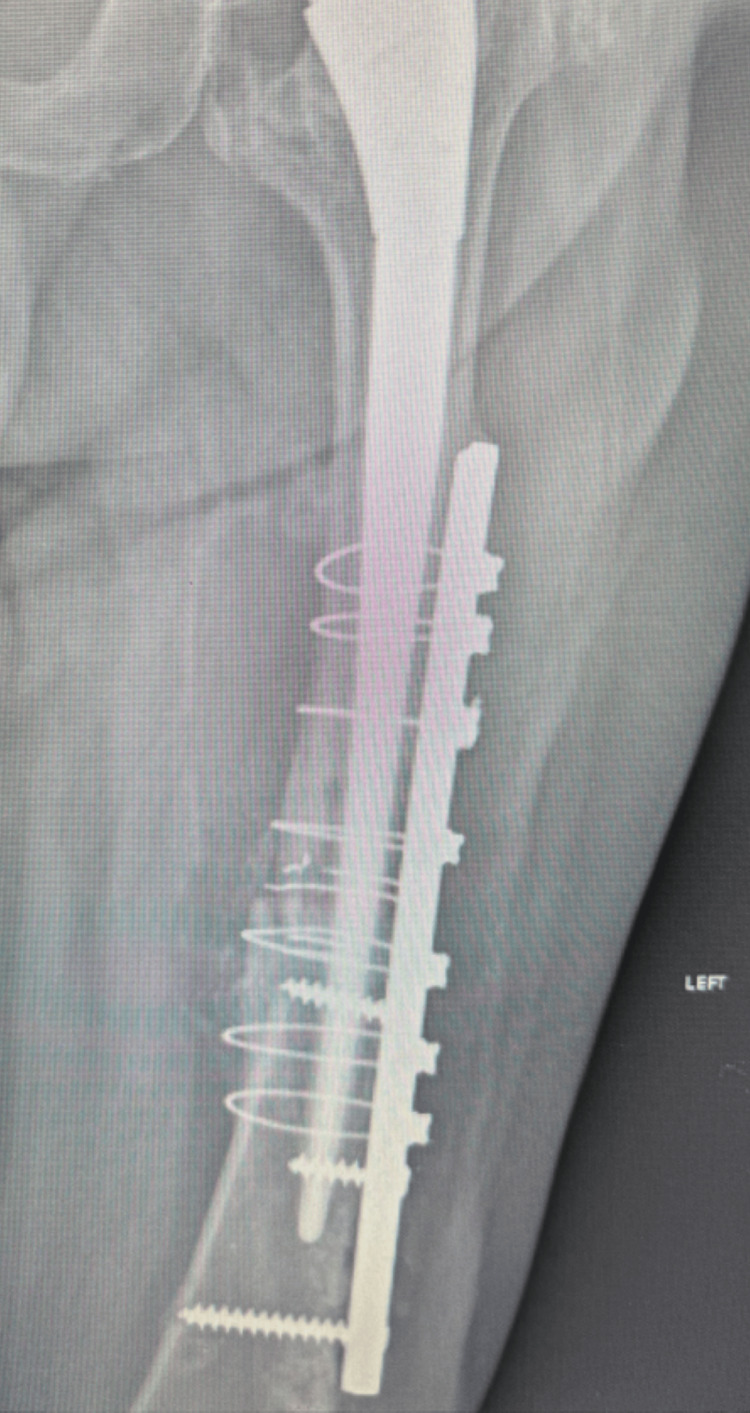
Postoperative radiograph demonstrating the final result following insertion of a long femoral stem for hemiarthroplasty and ORIF of the femoral osteotomy site using a lateral plate, screws, and cerclage wires ORIF: open reduction with internal fixation

The use of a high-speed pneumatic drill proved invaluable in facilitating the nail extraction. It effectively disrupted bone ingrowth and callus formation between the Kuntscher nail and the femoral cortex, enabling successful mobilization and removal. Throughout drilling, meticulous care was taken to minimize thermal injury by continuously irrigating the area with normal saline [4]. Following nail removal, the medullary canal and surgical field were thoroughly irrigated with normal saline to eliminate any potential metallic debris. The procedure then continued with hemiarthroplasty. A lateral hip approach was utilized, and a long femoral stem was implanted to bypass the osteotomy site. Stabilization of the osteotomy was achieved using a long lateral plate, screws, and cerclage wires (Figure 5). The duration of the surgery was approximately two and a half hours.

The patient was mobilized on the first postoperative day with non-weight-bearing and was discharged on postoperative day five. No wound-healing complications were observed. At the six-week follow-up, the patient was permitted to begin partial weight-bearing ambulation. Radiographic evaluation at six months demonstrated adequate healing of the osteotomy site. The most recent follow-up, conducted one year postoperatively, revealed evidence of heterotopic ossification on radiographs. However, the patient remained asymptomatic, with no reported pain or significant limitation in hip range of motion.

## Discussion

The Kuntscher nail is a rigid, cloverleaf-shaped intramedullary device that permits bone ingrowth into its recesses during fracture healing. While effective in stabilizing femoral shaft fractures, its design can render removal using closed techniques particularly challenging, often leading to failed attempts. Furthermore, repeated extraction efforts pose a significant risk of iatrogenic femoral fracture.

Various techniques have been described in the literature to facilitate the removal of retained Kuntscher nails [5]. Mari et al. [6] have reported a planned two-stage approach for a patient with hip arthritis and a retained Kuntscher nail placed 30 years prior for a femoral shaft fracture. Initial closed extraction attempts using a conical extractor and hook were unsuccessful. The authors then performed a large, unicortical one-third longitudinal osteotomy on the lateral femoral cortex, created a trench in the greater trochanter around the nail to access the proximal hole, and used an impactor engaged in the proximal hole to hammer the nail from below until the hole broke. Subsequently, they drilled a new hole distal to the damaged one using a diamond drill, which enabled successful nail extraction. Cerclage wires and a bone allograft were then used to reconstruct the osteotomy site. The hip arthroplasty was deferred as a second-stage procedure.

Randelli et al. [7] have described an innovative approach utilizing a custom-made, cannulated long trephine to ream the surrounding femoral bone directly around the Kuntscher nail. This technique enabled mobilization and extraction of the nail without the need for a cortical window or osteotomy. Although the method avoided the complications associated with open surgery, it required extensive preoperative planning, including a CT scan and the production of a patient-specific reamer. Georgiadis et al. [8] have reported a case in which a high-speed drill was used to create a channel within the intramedullary nail itself. A nail extraction hook was then inserted through the channel to facilitate removal.

Goosen et al. [9] have presented a unique case involving a primary total hip arthroplasty in a patient with a retained intramedullary nail. An extended trochanteric osteotomy was performed following femoral head resection. The proximal part of the nail was then transected with a high-speed diamond burr, and a cemented, small-diameter revision femoral stem was inserted, with its tip seated within the hollow proximal end of the retained nail. The osteotomy was then stabilized using cerclage wires. Nimberg et al. [10] have detailed a surgical technique for managing nail incarceration during intramedullary nailing by creating a cortical window approximately 5-7 cm in length at the narrowest segment of the femoral canal. Multiple drill holes were connected using an osteotome, and endosteal bone was removed with a chisel. This decompressive method enabled successful nail mobilization and removal. Although their context was intraoperative incarceration, the underlying principles are applicable to the extraction of a chronically retained Kuntscher nail.

Compared to previously described techniques, the approach used in our case offered several advantages. It allowed for single-stage management of both nail extraction and definitive treatment of the femoral neck fracture with hemiarthroplasty and osteotomy fixation. The use of a high-speed pneumatic drill proved effective in overcoming the limitations of closed extraction, without the need for patient-specific instrumentation or staged procedures. This method provides a practical solution when facing an incarcerated Kuntscher nail in complex revision scenarios.

Despite its effectiveness, the described technique for Kuntscher nail extraction carries several disadvantages. The procedure requires a larger surgical exposure due to the need for a transverse femoral osteotomy, resulting in a more extensive soft tissue dissection and consequently a bigger incision. This increases the risk of intraoperative blood loss and postoperative wound complications. Specialized equipment, such as a high-speed pneumatic drill, may not be readily available in all surgical settings and can increase the technical complexity of the operation. Additionally, the requirement for osteotomy fixation with plates, screws, and cerclage wires adds to the overall cost and duration of the surgery. However, despite these drawbacks, the technique becomes necessary in scenarios where the Kuntscher nail is firmly incarcerated and cannot be extracted using closed methods. In such cases, this approach provides a reliable and definitive solution to a challenging surgical problem.

This report also underscores a critical clinical principle: when encountering an incarcerated intramedullary device, surgeons must be prepared to employ flexible intraoperative strategies. Attempting prolonged closed extraction can increase operative time, blood loss, and risk of iatrogenic fractures.

## Conclusions

Although rarely encountered in contemporary orthopedic practice, the Kuntscher nail can present significant challenges when removal is required, particularly in cases of incarceration. Despite advances in surgical techniques and instrumentation, closed extraction methods frequently fail. Therefore, surgeons must be well-prepared with alternative strategies and appropriate tools. This case report highlights a complex yet effective technique for the successful removal of an incarcerated Kuntscher nail, demonstrating a viable option when conventional methods are insufficient.
